# Using of reciprocal teaching to enhance academic achievement: A systematic literature review

**DOI:** 10.1016/j.heliyon.2023.e18269

**Published:** 2023-07-14

**Authors:** Nofouz Mafarja, Mimi Mohaffyza Mohamad, Hutkemri Zulnaidi, Hidayah Mohd Fadzil

**Affiliations:** a Department of Education and Graduate Studies, Faculty of Technical and Vocational Education, Universiti Tun Hussein Onn Malaysia, Malaysia; b Department of Mathematics and Science Education, Faculty of Education, Universiti Malaya, Malaysia

**Keywords:** Reciprocal teaching, Academic achievement, Systematic literature review, Comprehension skills

## Abstract

Reciprocal Teaching a type of cooperative teaching and learning strategy involving learners with similar academic backgrounds sharing teacher and student roles. Its use was not explored to the same degree as other types of peer-assisted strategy, including students at different levels. The goal of the systematic review was to investigate the impact of Reciprocal Teaching on existing literature in the field of education which include the use of Reciprocal Teaching for all student's level to enhancing academic achievement and ROSES (Reporting Standards for Systematic Evidence Syntheses) was carried out as a review protocol to conduct Systematic Literature Review (SLR). A literature review performed between January 2013 and February 2022 using the relevant electronic databases (Eric Plus Text, PubMed, Web of Science, Scopus) as well as searching for the keywords. Twenty-eight researches met the set inclusion requirements for this review. The results found that Reciprocal Teaching improved comprehension of the subject under review, enhanced cooperative learning, improved academic achievement, communication, metacognition, teaching skills, developed a positive impact in learners understanding, especially in evaluating learning performance and reciprocal teaching which may improve the understanding of reading, solving word problems in scientific fields.

## 1 Introduction

Reciprocal teaching, also known as reciprocal learning, is a strategy that puts the students in charge of the content [1,2]. Instead of the teacher reading to the students or having them read out loud during class time, the students are given independent reading time to pursue the material, then given time to discuss what they have read [3]. This type of strategy puts the onus on the students, having them use their minds to take in the content. Then, the teacher helps them start a discussion about the text where they can ask clarifying questions and analyze what they've read.

Reciprocal teaching allows students to come together to and encourages the sharing of information or the completion of instructional task success procedures in collaboration. The concept of cooperation is the foundation of the point of view and should be understood. Palincsar and Brown [4] addressed a reciprocal teaching aimed at equipping topics with tools to facilitate the use of metacognition and reading comprehension approaches. Reciprocal Teaching is the name of their program. “ Reciprocal Teaching is a" learning technique in which comprehension of reading is seen as an operation of problem-solving that facilitates thought while reading " [2,5]. During a small group reading session, fluent readers can benefit from Reciprocal Teaching to improve their comprehension skills.

Teachers can use reciprocal teaching components as a variation on a guided reading session, because there is less teacher involvement and more student independence in this practise, students must read more independently than in a structured guided reading session [2,[6], [7], [8], [9]]. The reciprocal teaching or learning is a form of teaching that assists the instructor. Reciprocal Teaching is an interactive method designed to improve students' comprehension skills and to foster metacognitive habits, which are defined as thinking about thinking, recognising what one knows and what one does not, as well as organisational strategies for managing the process. Referring to Oczkus [10], Peer Learning Reciprocal Teaching describes four predictive roles, predictor, clarifier, questioner, and summarizer, making it a helpful technique to improve learning difficulties such as: listening, speaking, understanding, decoding, phonetic knowledge, word recognition, comprehension, computation, and problem solving. Palincsar and Brown [4] were the first to use reciprocal teaching to overcome low attention distress, as it provides an interactive environment that engages every student, in which they are exposed to low attention span negatives. A specific, essential role is assigned to mitigate the effects and create a functional environment. Researchers who developed reciprocal teaching, Palincsar and Brown [4], discovered that after using the tactics with a sample of students for just 15–20 days, assessments of their reading comprehension rose from 30% to 70%, and there is evidence that students who use this strategy are more likely to venture outside of their study subjects. Reciprocal teaching strategy strengthened by using four elements (predicting, questioning, clarifying, and summarising). According to Mehmood and Alvi [8] reciprocal teaching enhances students' confidence and persuades them to learn, which in turn improves understanding and academic performance alongside an awareness of weaknesses in areas where they cannot read. Philippa [11] suggested that teachers should use reciprocal teaching as a teaching method if students can read the text, but do not understand what it means; using reciprocal teaching with students will help to improve comprehension skills (word problems) and critical thinking. Reciprocal Teaching is considered a scientific method, but this method is not properly practiced with scientific filed. Some researchers focus on four stages or strategies of reciprocal teaching: i.e., predicting, clarifying, questioning, and summarising [3,[6], [7], [8], [9],[12], [13], [14], [15], [16], [17], [18], [19], [20], [21], [22], [23], [24], [25], [26], [27], [28], [29], [30], [31], [32], [33]]. However, other researchers have concentrated on the eight stages or techniques of reciprocal teaching, including anticipating, clarifying, questioning, picturing, connecting, calculating, summarising, and providing feedback [34]. Reciprocal teaching model can be used to enhance students' cognitive development and retention in all school and university levels. Reciprocal teaching is a supportive teaching method because it fosters meaningful student dialogue, including extended discussion of texts, and helps students hone their skills in finding, recording, and organising information in preparation for writing [25]. Additionally, Reciprocal Teaching clarifies what readers predict, explain, query, and summarize; it also aids students in developing comprehension methods in a supportive atmosphere; and it broadens their subject-matter vocabulary [21]. Students have the opportunity to debate their ideas with their group members while participating in reciprocal teaching [6]. Although they frequently approach the teacher for help, they prefer to talk about issues with their classmates and pose questions to themselves and to each other. That is, children receive assistance from both their classmates and their teachers in developing their questioning, arguing, and validating skills. Students may gain confidence in their capacity to answer word problems and expand on prior knowledge by employing a reciprocal method in any subject, such as math teaching, by paying attention to the ideas and information of other students in their groups [26]. However, reciprocal teaching helps students to improve their comprehension of text by teaching them how to predict, question, clarify, and summarize information. This approach helps students to engage with the text and develop a deeper understanding of the material. Reciprocal teaching helps students to develop critical thinking skills by teaching them how to analyze and evaluate information. By predicting, questioning, clarifying, and summarising information, students learn how to think critically about the text and develop their own perspectives and opinions. Reciprocal teaching promotes active learning by encouraging students to participate in the learning process. By engaging in the four key strategies of reciprocal teaching, students become active learners, rather than passive recipients of information [29]. Reciprocal teaching supports differentiated instruction by allowing teachers to tailor the instruction to the needs of individual students. By providing students with different reading materials and allowing them to work in small groups, teachers are able to differentiate instruction to meet the needs of all learners. Reciprocal teaching increases student motivation by providing students with a sense of ownership over their learning [25]. By engaging in the four key strategies of reciprocal teaching, students feel more invested in the learning process and are more motivated to continue learning. Reciprocal teaching encourages collaboration by requiring students to work in small groups and share their ideas and perspectives with each other. This approach helps students to develop their social and emotional skills, such as communication, collaboration, and empathy [33].

Overall, reciprocal teaching is a structured approach to teaching that has many benefits for both students and teachers. By improving comprehension, developing critical thinking skills, promoting active learning, supporting differentiated instruction, increasing student motivation, and encouraging collaboration, reciprocal teaching helps to create a more engaging and effective learning environment.

Academic achievement is defined as performance outcomes that show how well a person performed against specific objectives that were the focus of classroom activities, particularly in school, college, and university (critical thinking) or the acquisition of knowledge and understanding in a particular intellectual area (eg, arithmetic, writing, science, history) [35]. The definition of academic success depends on the metrics used to quantify it because it covers a wide variety of academic outcomes. Academic achievement can be determined by a variety of factors, including procedural and declarative information gained through formal schooling, curriculum-based factors like grades or results on academic performance tests, and cumulative indicators for academic services and qualifications [21].

Academic achievement among students is a hot topic because of how important it is. It displays how well children have mastered fundamental skills and also shows when learning has taken place. Academic achievement is the calibre of a student's performance in the classroom. It has to do with the pupils' skills as well as their understanding of a subject that they studied in school [36]. Academic achievement, according to Ref. [18], is the mastery of fundamental ideas and concepts, significant truths, practical abilities, strategic knowledge, and the integration of information. The status of subject-matter knowledge, comprehension, and abilities over a predetermined time frame or the levels that pupils have advanced in all academic content areas are also included. It also shows how capable pupils are at finishing assignments and studying [37]. Academic achievement is the outcome of a learning process that measures how well students, teachers, or institutions accomplish their educational objectives [38]. Literature reviews are essential for: (a) determining what has been written on a subject or topic; (b) determining the extent to which a specific research area reveals any interpretable trends or patterns; (c) aggregating empirical findings related to a narrow research question to support evidence-based practise; (d) generating new frameworks and theories; and (e) identifying topics or questions requiring further investigation.

The purpose of this review is to examine the effectiveness of reciprocal teaching as a reading comprehension strategy for school students. Specifically, this review will focus on experimental, quasi-experimental, qualitative method and mixed method studies published in the last 10 years that have assessed the impact of reciprocal teaching on academic achievement, as measured by standardized tests or teacher-made assessments. The review also will focus on young, adult and students with disabilities because young and adults may face unique challenges in their education as they transition from high school to college or the workforce, and may benefit from targeted interventions like reciprocal teaching to improve their reading comprehension skills. Similarly, the introduction could explain that students with disabilities may face additional barriers to learning and may require specialised interventions to support their educational success. The review will also consider the Subject design, Participants characteristics, Theories, Applications of reciprocal teaching, and Advantages. Having a well-defined scope helps to ensure that the review is focused and relevant to the research question, and it makes it easier for readers to understand the purpose and scope of the review.

### 1.1 Objectives

This systematic review paper aim to examine the impact of Reciprocal Teaching on existing literature in the field of education which include the use of Reciprocal Teaching for all student's level to enhancing academic achievement and understanding concepts (words problem solving). The second aim is to propose evidence-based guidelines on the consequences of future studies and the implementation of a reciprocal teaching strategy. This study is conducted with two research questions in mind.

1. What are the effects of using reciprocal teaching strategy on students' academic achievement?
2. What are the significant challenges did teachers face in using reciprocal teaching?

## 2 Materials and methods

### 2.1 Research design

Systematic Literature Review (SLR) was conducted using the Reporting Standards for Systematic Evidence Syntheses (ROSES) procedure [39]. ROSES was developed with the goal of ensuring and managing the review's quality while also enhancing and maintaining a good method for developing an SLR through more openness. The review methodology is appropriate because it was developed to take into account the complexities and variability across various scenarios and studies surrounding the synthesis approach, even though ROSES was specifically developed for environment management and this review is primarily focused on disaster management [39]. The next step was to create and carry out the strategy for document searching through three organized steps: identification, screening, and eligibility. Following that, a quality evaluation procedure was carried out using the modified criteria [40]. Here, the calibre of each chosen item was evaluated before its inclusion in the review. The chosen papers were then put through several steps, including data extraction and data analysis. To make sure the review process served the review's purpose, the authors followed the recommendations made in the review where it was appropriate by taking into account alternatives. As a limit of time period and language is required when producing a systematic review study, reports from January 2013 to February 2022 were searched within a historical span of just over 10 years.

### 2.2 Search strategy

The following steps or techniques are required when creating a comprehensive search strategy for a systematic review: formulate the research question, identify key concepts, and research the literature Make up search phrases, such as free-text ones, create search words like wildcards and proximity operators, search fields, phrase searching, search restrictions, and so forth. Pilot a search plan and monitor its development change the search syntax for different databases and final search strategy to look for computerised databases or other resources, there are several examples, including Eric Plus Text, PubMed, Web of Science, Scopus Research Databases: The main search words used were: Reciprocal teaching, Reciprocal Peer Instruction, reading comprehension, academic achievement and learning outcomes.

### 2.3 Eligibility

**Fig. 1:**
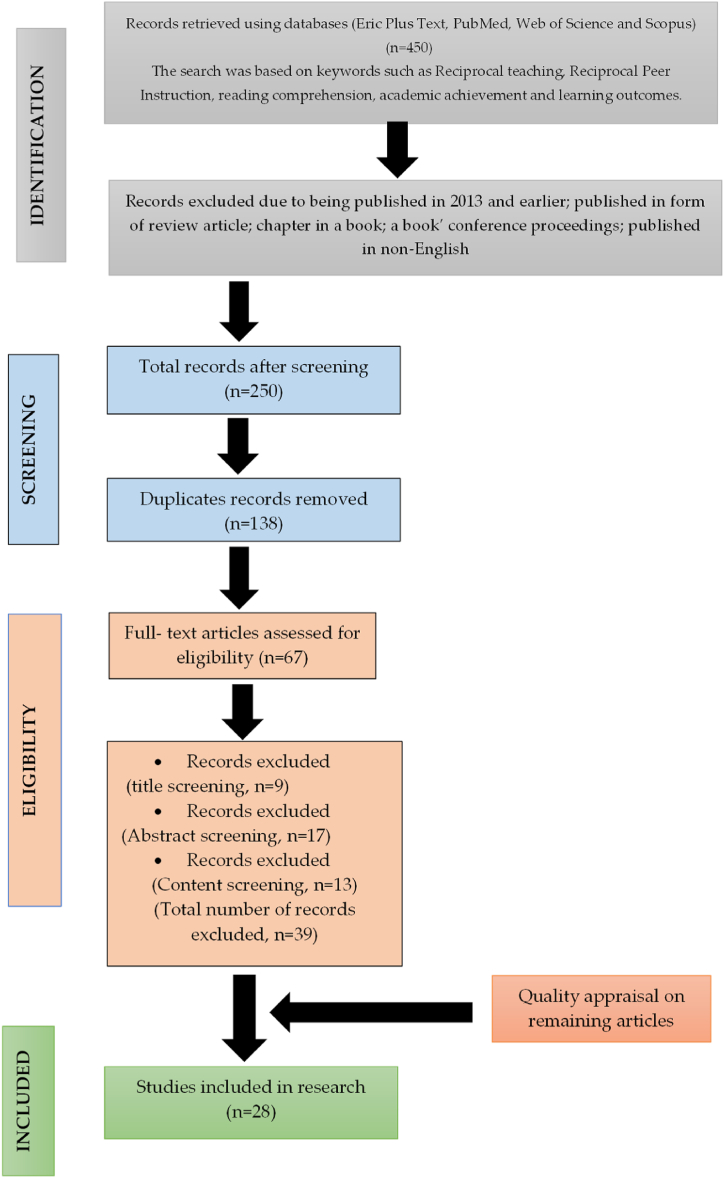
Systematic searching data.

Partway through this stage, authors manually reviewed the remaining manuscripts to determine which met the predetermined inclusion criteria (by reading the title, abstract, or full paper), as recommended by Ref. [41] First of all, 9 articles were eliminated during the title screening stage, while 17 articles were eliminated during the abstract screening stage. After reading the content of the selected articles, the authors excluded another 11 articles. At this point, 39 articles were removed because they did not concentrate on reciprocal teaching with academic achievement and were also in the form of a review paper. The total number of articles submitted for the quality assessment step was 28. (See Fig. 1).

### 2.4 Inclusion and exclusion criteria

Insertion criteria included teaching space and research laboratories published in English and Arabic between January 2013 and February 2022 for students in primary, and secondary schools' undergraduate students and teachers. The way in which the effects of reciprocal instruction on people's perception of reading are analyzed to learn measures and other variables such as academic achievement. Study was to include expert scaffolding, as well as the four techniques of Palincsar and Brown to enhance academic achievement. The exclusion criteria included the research who's studied reciprocal teaching with other variables excepted academic achievement, learning outcomes, performance and reading comprehension skills variables.

### 2.5 Data extraction and analyses

This section explains the data extraction procedure, which is used to determine the rigour of the approach, as well as the analysis utilised in selected publications. Because the evaluation depended on a variety of study approaches, as recommended by Whittemore and Knafl [42], the publications were typically thematically examined to provide the most useful strategies for reconciling the disparities. Furthermore, thematic analysis is a sort of research that looks for patterns in prior studies by evaluating any parallels or connections that may exist in the data. The current review was based on the method advocated by Flemming and Briggs [43], who emphasized the importance of theme synthesis in synthesising data from varied study designs due to its adaptive mode despite the possibility of performing several qualitative syntheses. These approaches, as recommended by Kiger and Varpio [44], were employed as the foundation for the topic synthesis in this work.

## 3 Results and discussion

### 3.1 Overview of studies

Study outline 67 papers were known in higher and lower Education on the concept of combining the most used search key words. Once narrative reports, review paper papers, and non-peer-reviewed journal papers were excluded, 28 qualifying studies were listed. To make sure they complied with the inclusion and exclusion criteria, the abstracts and complete papers were then reviewed. A fastidious analysis of the definition of Reciprocal Teaching culminated in the removal of thirty-nine studies with non-conforming meanings, reflecting a final assortment of 28 publications to be included. There were 2 studies that used mixed approaches and 26 quantitative. The limited number of papers resulting from this study shows that Reciprocal Teaching has not been widely studied in recent years, most of the researchers focus on reciprocal teaching to enhance critical thinking skills on Science subjects.

### 3.2 Study designs

Questionnaires, assessments, test surveys and focus group interviews were not used to collect qualitative and quantitative data, as shown in Table 1, twenty-eight studies [3,[6], [7], [8], [9],[12], [13], [14], [15], [16], [17], [18], [19], [20], [21], [22], [23], [24], [25], [26], [27], [28], [29], [30], [31], [32], [33],[45], [46], [47], [48], [49],^50^]. Accessed students’ course grades from different levels. Quantitative information was also gathered using the teacher intervention profile, Likert Scales, assessment checklist, and academic scores. Some people used video recording observations to gauge performance success rates.

**Table 1:** Description of the selected studies.

No	Author, Year, place of Study	Study design	Sample	Theory used	Study aim	Reference
1	Gilbert Banguis Guita and Denis Abao Tan, 2018, Philippines	quasi- experimental research design	Magpet National High School is located in the Poblacion of Magpet, North Cotabato. Students in Grade 8 were divided into two intact classes. Each of the chosen sections had 38 students.	Lev Vygotsky's scaffolding theory (1978)	determined the academic progress and mathematical anxiety of students in a cooperative learning setting.	[3]
2	Amal Mohamed Falah Al-Saraireh, 2013, Jordan	experimental research design	There were 53 students in total, with 26 female students in the control group and 27 female students in the experimental group.	Cognitive theory	Create a curriculum based on the reciprocal teaching approach, and evaluate its success in improving academic performance and fostering geographic problem-solving skills in Jordanian sixth graders.	[6]
3	Sumaera Mehmood and Muhammad Mushtaq Alvi, 2017, Pakistan	The Pre & Post equivalent group design	The experimental (n = 33) and control (n = 32) groups	scaffolding and facilitating meta cognition	Examine the impact of traditional versus reciprocal teaching on the academic performance of secondary students.	[8]
4	Zaman, 2019, Pakistan	Two groups were quasi-experimental design; one experimental group and the other control group.	The sample consisted of 118 students from four randomly selected schools.	Cognitive theory	To explore the impact of Reciprocal Teaching (RT) on the progress of students at secondary level in achievement physics.	[32]
5	Tun Zaw Oo, Andrea Magyar and · Anita Habók, 2021, Myanmar	Two groups were experimental design; one experimental group and the other control group.	Every ninth-grade student from the five basic education upper secondary schools chosen	cognitivist, and constructivist theory with Vygotsky's social development theory	examines how well Myanmar upper secondary school students in English reading comprehension achievement is affected by the reflection-based reciprocal teaching (RBRT) approach.	[7]
6	Kiran Dadabhoy, b Marium Dadabhoy, 2021, Pakistan	mixed methods study	30 secondary students of grade 11	cognitive and metacognitive theory	Improving Secondary Students' Reading Skills and Academic Performance	[9]
7	Agoro, Akinsola, 2013, Ibadan, Nigeria.	The pretest-post-test, control group, quasi-experimental research design was adopted for this research.	(294) pre-service science teachers at the high, medium, and low levels The sample was designed to assess numerical ability.	meta-cognition theory	To examine how pre-service teachers' achievement and scientific method skills differ while receiving reflective-reciprocal education versus receiving it from a peer tutor.	[13]
8	Leonardi Jaye Putra, 2021, Indonesia	Experimental Design	60 female students	cognitive theory	Examine the impact of reciprocal instruction on the reading comprehension performance of female students.	[14]
9	Capanzana, Avilla, 2017. Lopez, Quezon, Philippine	quasi-experimental pretest-posttest research	(131) ninth-grade students from four separate sections of a public high school in Lopez,	Vygotsky's “zone of proximal development.” “proleptic” teaching and Vygotsky's social development theory includes expert scaffolding.	to look at how ninth-grade chemistry students' reading comprehension, academic performance, and self-regulation are affected by the Reciprocal Teaching method with Self-Regulated Learning (RT-SRL).	[25]
10	YEN-JU HOU, 2015, Taiwan	Quasi- experimental design.	There were 107 students in total, with 77 in the experimental group and 30 in the control group.	Metacognitive theory	Find out how reciprocal teaching (RT) affects metacognitive awareness and reading comprehension achievement in junior college students.	[15]
11	Ogunyebi, Tunji Henry, 2018, Nigeria	Quasi- experimental design.	120 junior secondary school students were chosen using a multistage random sampling technique.	cognition theory.	Effects of a reciprocal instructional technique on Basic Science performance in Ekiti State junior secondary school students	[16]
12	Abdul Ameer, 2017, Iraq.	Quasi- experimental design.	60 female students were selected from intermediate schools.	Cognitive theory.	determine how adopting an exchange technique affects students' achievement in teaching Biology for female second-year intermediate students	[12]
13	AL Shshaw, 2018, Iraq.	Quasi- experimental design.	56 students from faculty of Education.	Metacognition theory.	The impact of reciprocal teaching on academic performance and the growth of cognitive metacognition abilities.	[20]
14	Siti Zubaidah, Susriyati Mahanal, Mar'atus Sholihah, Fatia Rosyida and Zenia Lutfi Kurniawati, 2020, Indonesia	Quasi- experimental design.	Natural Science classes with 125 tenth-grade students.	Cognitive theory.	Analyze how the Remap RT (Reading - Concept Mapping - Reciprocal Teaching) learning model affects the biology performance of students with limited ability.	[18]
15	U. A. Ginga, B. Mohammed & N. Usman, 2019, Nigeria	quasi-experiment research.	210 students' were selected using simple random sampling technique and participated in the study.	To improve reading comprehension, based on Vygotsky's kind of reading technique teaching and achievement	how a reciprocal teaching approach affected students' performance on a math word problem	[19]
16	Alemu, 2020, Ethiopia.	quasi-experiment study.	The total number of students has now reached 126.	Language-mediated social construction of knowledge and the concept of zone of proximal development (ZPD).	To ascertain the most efficient mix of direct teaching techniques and reciprocal peer tutoring for raising student achievement in secondary school physics instruction in a content-focused and exam-oriented curriculum.	[17]
17	Komariah, Ramadhona, Silviyanti, 2015, Indonesia	Quantitative and qualitative research.	The subjects were 24 twelfth-grade students from a high school in Banda Aceh.	Cognitive theory.	To determine students' achievements following four comprehension training sessions using RTM.	[22].
18	Mulbar, Zaki, Nurwahidah, 2019, Indonesia	quasi experiment research	12 UNM Mathematics Department students in a capital market mathematics class.	Cognitive theory.	to ascertain the reciprocal teaching model's influence on academic achievement in higher education.	[23]
19	AlSaraireh, Ku Hamid, 2016, Jourdan	quasi experiment research	176 participants participated and were randomly chosen and split into two mixed gender groups.	Cognitive theory.	To determine the effects of using the reciprocal teaching paradigm on first-year Jordanian students' reading comprehension performance at Mutah University.	[49].
20	Erwanto, Maryatmi and Budiyanto, 2018, Indonesia.	Level 2 treatment was used in the experimental research.	48 students divided into four groups 2 groups reciprocal teaching with high and low self-efficacy other 2 groups Expository learning.	Metacognitive theory	to gain knowledge on the effects of reciprocal teaching and self-efficacy on the results of AUD mathematical logic learning.	[48]
21	Bilgoon, 2017, Saudi Arabia.	Experimental design	80 students were chosen at random from the fifth primary level.	Metacognitive theory	to assess, using a sample of talented primary school pupils engaged in a science course, the efficacy of reciprocal teaching in fostering meta-scientific reading abilities and academic accomplishment.	[47]
22	Affiana Muthik, Arif Muchyidin, Alif Ringga Persada, 2022, Indonesia	Quantitative method	136 students	Constructivism theory	examined the impact of students' learning motivation on learning outcomes and the reciprocal teaching-learning model.	[21]
23	Samar Abdul – Aziz Al – Shalhoub, 2013, Saudi Arabia	quasi experiment research	The experimental group consisted of (115) students, while the control group consisted of (128) students.	Constructivism theory and cognitive theory.	determined the reciprocal teaching on mathematics teaching, mathematical communication development, and academic achievement	[24]
24	Muhamad Abas, Etin Solihatin, Nadiroh, 2019, Indonesia	Quasi experimental method,	92 students from Halu Oleo University	Cognitive theory	to find out how interpersonal quotient and educational outcomes for students interact.	[26]
25	Novy Yuliyanti, Siti Hikmah and Masrupi, 2018, Indonesia	quasi experimental research	One class was designated as the experimental class, and the other as the controlled class, both of which had 30 students.	Cognitive theory	to look into the effects of a reading habit and a reciprocal teaching technique on the reading comprehension of MTs Al-Hasyimiyah eighth graders.	[27]
26	Jubeir Suleiman Samir Al-Harby, 2016, Saudi Arabia	quasi-experimental method	primary 4Th and 5Th grade	Cognitive theory and constructivism theory	The primary objective of the current study was to assess the impact of the reciprocal-teaching technique on the academic performance and cultural attitudes of Qassim University students.	[28]
27	Jimoh Bakare & Chibueze Tobias Orji, 2018,Nigeria	quasi-experimental research design.	107 s-year bachelor's degree students (sophomores) from Lagos State's vocational and technical education department	Cognitive theory and constructivism theory	Analysing the results of direct learning environments and reciprocal peer tutoring on sophomore academic performance in Nigerian electrical and computer basics	[29]
28	Rusli, M., Degeng, N. S., Setyosari, P., & Sulton. (2021). Indonesia	quasi-experimental research design.	The sample was 56 students (25 females and 31 males) who were enrolled in a Homiletics course at the Southeast Asia Bible Seminary (SAAT) in Malang, Indonesia.	Bandura's social learning theory, Piaget's cognitive development theory, and Vygotsky's social constructivist learning theory	empirical study aims to verify whether peer teaching is effective when applied in a theological school setting	[50]

### 3.3 Participant characteristics

The majority of the studies were conducted with undergraduate students, teachers, pre-service teachers, and secondary level students, with five studies selecting first year students, two studies selecting third years, and one study selecting high school students. One article was identified from Myanmar, two from Jordan, three from Pakistan, two from Iraq, eight from Indonesia, five from Nigeria, three from Saudi Arabia, one from Ethiopia, one from Taiwan, and two from Philippine. The disciplines included mathematics, language, science literacy, Islamic studies, Physics, biology, chemistry, electronic and computer fundamentals (vocational education) and economics.

### 3.4 Theories and aims

Education was included as a majority of the disciplines in this review because it involves studying psychology, learning outcomes, and theoretically (mathematics, language, science literacy, Islamic studies, Physics, biology, chemistry and economics). The majority of the studies used theories as a framework to support their research. One study combined Vygotsky's social development theory and cognitive development theory, on the other hand, the other three studies only used Vygotsky's social development theory, while most of researchers used cognitive theory [12,21,28,31,34] five study used metacognition theory [13,15,16,20,22,23,25,26], three studies used social constructivism theory and cognitive development theory, however; one study used constructivism theory, and three studies used constructivism theory with cognitive theory and one study used cognitive with metacognition theories, two researchers used social constructivism theory with Vygotsky's social development theory. Study aims to improved academic performance and learning outcomes, investigate reciprocal teaching as a teaching method, its application in the majority of teaching environments, teaching acquisition, and its impact on communication skills, academic achievement and reading comprehension skills in the most fields or subjects.

## 4 Using of reciprocal teaching strategy

Reciprocal Teaching was chosen by the majority of researchers given that it places a strong premium on reading comprehension, especially in the short term, and because it has a clear goal and aim, as well as a plethora of favourable research data. Students are taught four techniques in this programme for improving their ability to read comprehension and read comprehension while self-monitoring [4]. Posing questions, summarising, clarifying, and forecasting are the four strategies. Reciprocal teaching has been commended for its ability to support students in improving their reading abilities in pre-post trials or research studies. Reciprocal teaching enables beginning readers to pick up and internalise the techniques used by proficient readers. The novices are practising and improving the abilities needed to understand and learn when they use reciprocal teaching tactics. Additionally, research using the reciprocal teaching method has repeatedly shown that it improves reading comprehension as seen on standardized reading tests. The method was reportedly tested by one of the researchers, Palinscar, in a range of contexts, including (1) one-on-one tutorials, (2) small-group sessions led by trained reading specialists, (3) small-group sessions instructed by general classroom teachers without specialised training, (4) whole-group instruction in the method by teachers without specialised training, and (5) small-group discussions moderated by peers of the group members. In every instance, student comprehension and academic achievement increased—even in the groups that the students led. We considered the method to be the best because it offered a variety of opportunities for introducing and reinforcing the techniques. We reasoned that it would be far simpler to get the teachers on board than to expect them to learn a whole new paradigm. Because this method is simple to understand, we were confident that it would give us a model we could use to instruct parents (and volunteers) on how to support promoting comprehension among their children and thereby reinforce reading skills that would help students develop their academic achievement and other skills [51]. This was true for teachers as well as students, regardless of the level of training in reading research and applications (or even ability to read). According to the new definition of reading, reading is an interactive process in which readers engage with the text while drawing on their existing knowledge, is where Reciprocal Teaching most closely matches with the definition. By drawing on prior knowledge, readers can discover new facts, fundamental concepts, and points of contention. Most importantly, readers create meaning from the text by paralleling, contrasting, or affirming what the author suggests. This is a structure that all excellent readers employ. Otherwise, the text would just be a bunch of random letters on the page. Learning cannot take place if meaning is not constructed. Reciprocal Teaching is a cooperative and constructivist learning model [33]. Four comprehension techniques are used in the reciprocal teaching method: predicting, questioning, clarifying, and summarising. These methods assist students in keeping track of their independent reading comprehension growth. Students are grouped into teams of four or five. A group will be divided into five positions, according to Palinscar and Brown [4], namely the leader, predictor, clarifier, questioner, and summarizer. Additionally, the reciprocal teaching method can be used to teach pupils from elementary school to university-level mathematics and physics in addition to English [34,52]. This approach was created to teach students how to use the comprehension-related methods.

## 5 Advantages gained from reciprocal teaching strategy

Reciprocal teaching is a great way to teach students how to recognise valuable reading ideas while learning about vocabulary, generating ideas and questions, and summarising data. It can be used in many areas of content; it works especially well for textbooks and non-fiction texts. While there are many advantages to using a reciprocal teaching approach, including: Increasing student comprehension of the material, this benefit of reciprocal teaching is most readily apparent when it comes to how well kids read and comprehend. While many kids can read, they frequently struggle to understand what they have read. As a result, teachers can utilise students' reading and comprehension skills by using the reciprocal teaching techniques. Second, students develop their capacity for metacognition. The ability to critically evaluate one's own assumptions and decide if one is right or wrong while reading is another intriguing benefit of reciprocal instruction. Third, because it is immersive, reciprocal teaching promotes students' active involvement in the learning process. It is a great approach to get kids involved in the learning process. Students also take the lead in group conversations, serve as teachers, ask questions, and answer those questions. Fourth, it promotes group learning. The majority of reciprocal teaching takes place in groups. The teacher splits the class into groups so that each group can talk, take turns reading the material, and explain its significance. Students assume the role of a teacher or reader, and as they read the book, other students quiz the reader. Determined by the student's response, the reader's understanding of the material is ascertained. In addition, students analyze texts both individually and in groups to discern their intended meaning. Fifth, students increase their vocabulary knowledge. When students come across unfamiliar words in the text and need more understanding and clarity, teachers frequently allow them to consult dictionaries during the clarifying stage of reciprocal teaching. Students are constantly learning new words and meanings that they can use when speaking or writing as a result of this. They can also use their vocabulary knowledge to better understand text. Sixth, Reciprocal learning is advantageous for students who have learning impairments [52]. High-achieving students might be able to read and understand text without the aid of reciprocal education. On the other hand, those who are slow learners or have learning difficulties may benefit from the procedure. The reciprocal process is incredibly strong and useful, starting with the prediction stage, where students must anticipate the subsequent events in a passage before reading it, and continuing with constant questioning of the reader to gauge their understanding of the text, clarification of unclear passages, and summary. As a result, it is an effective tactic that enables teachers to devote more time to assisting readers in understanding. The reciprocal teaching paradigm takes into account the fact that children with learning difficulties, in particular, need more care, patience, and time. Improved communication, better teaching abilities, independent learning and problem solving, and collaborative learning and working are the main transferrable skills taught through the use of Reciprocal Teaching [14,17,19,29,32]. Among the discipline-specific benefits were improved understanding and retention of the topic [8,16,20,30]. Increased knowledge and skills, improved course grades, and self-directed learners Student scores did not differ between peer-led and faculty-led groups [15,24,51,52]. The claims that Reciprocal Teaching improved conceptual understanding and communication skills were based solely on student perspectives, with no support for objective measures. Increased median scores as a result of Reciprocal Teaching were discovered by comparing grades of students who had experienced Reciprocal Teaching with those who had been traditionally taught the previous year [45,50]. While these researchers admit that their study design was limited, they failed to recognise that variables such as psychosocial factors could have an impact on student academic performance [46]. Some arguments about the effectiveness of Reciprocal Teaching may be questionable because a wide range of factors that could influence the results were not considered. The majority of the studies relied on both student and academic staff perspectives and did not measure objective changes as a result of the Reciprocal Teaching. More research is needed to objectively measure changes in actual learning. However, there was some advantages of using reciprocal teaching with students such as: enhances student engagement in the learning process, encourages collaborative learning, helps students develop their vocabulary knowledge, improves academic performance in the majority of fields or courses, and caters to students with learning difficulties through reciprocal learning.

## 6 Discussion

The goal of this systematic review was to explore the learning and teaching benefits and challenges of reciprocal teaching in improvement of academic achievement. It jointly aimed to explore but reciprocal teaching in several topics, such as: English language, Arabic language, mathematics, science subjects, and health education, can be applied with success across all student levels. One motivation for the use of Reciprocal Teaching has been to increase student numbers plus dispersed teaching tools, yet to jointly experiment with alternative benefits such as leadership development, teaching abilities and professionalism. This analysis also discovered that Reciprocal Teaching aided in achieving metacognitive control, it may be characterised as a group of self-control abilities utilised to effectively coordinate self-learning [25]. Recent findings according to De Backer et al. [50] further support Reciprocal Teaching potential as a metacognitive control marketing strategy. In this study, first-year undergraduate academic science students participated in eight Reciprocal Teaching sessions that were conducted in a quasi-experimental pre-test post-test format. The live learners' capacity for metacognition was habituated to through think-aloud and spoken protocols. Increased metacognition of scholars via Reciprocal Teaching was found in their results. Metacognition is seen to be critical for the growth of tutorial life readiness and capability [45], as well as for career development [53]. It has been emphasized [47] that it has not been formally incorporated into educational curriculum levels, despite numerous recorded benefits of different peer-assisted learning types. In previous Reciprocal Teaching studies, the relationship between techniques was simpler in terms of study understanding or frequency suggested, seemed questionable because they did not meet standards compare the effectiveness of techniques, including confirming or withdrawing the prediction, clarification and wonder about one's understanding and drafting review the summary. Similarly, believe that evaluating strategies is unnecessary because the effectiveness of using approaches in reciprocal education has given learners confidence in their reading dreams and wishes. Second, previous research has only addressed the students' reading process in recognising and resolving their reading difficulties. It was rarely discovered from process data to specify how: students confirm or refute their previous predictions, as well as keywords and topic phrases chosen and reselected by them, and to outline a summary and adjust how they used what they had figured out in a note. This vital information was never returned to either the teacher or the individual student. Because lack of teacher's ability to observe the students' reading procedures in detail, the teacher is unable to identify their reading difficulties. As a result, the teacher had little guidance for providing adequate scaffolding. Similarly, students cannot control and regulate their own reading processes.

Students with disabilities may face additional barriers to learning, which can impact their reading comprehension abilities. Reciprocal teaching can be a particularly effective tool for supporting these students, as it provides a structured and supportive environment for learning. For example, a teacher might use reciprocal teaching to help a student with a learning disability improve their reading comprehension. The teacher could model the strategy of predicting by reading a passage aloud and asking the student to make predictions about what might happen next. The teacher could then model clarifying by explaining any confusing vocabulary or concepts in the passage, and questioning by asking the student to generate questions about the text. Finally, the teacher could model summarising by asking the student to summarize the main points of the passage [54].

This process of reciprocal teaching can help students with disabilities to become more engaged in their learning and to develop stronger reading comprehension skills. By actively participating in the discussion and practicing the four key strategies of reciprocal teaching, students can learn to monitor their own understanding of the text and develop strategies for overcoming any barriers to comprehension. Moreover, reciprocal teaching can be adapted to meet the unique needs of students with disabilities. For example, a teacher might use visual aids or assistive technologies to support students with visual or auditory impairments. By tailoring the approach to the individual needs of each student, teachers can help to ensure that all students have the opportunity to develop strong reading comprehension skills (Takala, 2006) [55].

## 7 Conclusion

The review has explored used of reciprocal teaching strategy among reading comprehension, language skills, mathematics, chemistry, biology and general science, [27,30]. Discipline-specific and general edges will be obtained through this educational method. Careful designing and preparation for teaching roles is important to make sure that participants have a positive effect with reciprocal teaching and learning expertise regarding Reciprocal Teaching [3,[6], [7], [8], [9],[12], [13], [14], [15], [16], [17], [18], [19], [20], [21], [22], [23], [24], [25], [26], [27], [28], [29], [30], [31], [32],^52^]. Taking under consideration the annual levels of the participants is additionally beneficial for optimum outcomes. Though students are active participants, the academic facility is helpful for guaranteeing continuous support and compliance, particularly if participants are unaccustomed this educational project. Reciprocal Teaching remains a promising instructional tool, unfinished investigation in tertiary in educational programs. This study provides a thorough examination of the evidence on reading comprehension interventions for students. Even though many studies have shown the effect of reciprocal teaching in increasing reading outcomes and academic achievement [3,[6], [7], [8], [9],[12], [13], [14], [15], [16], [17], [18], [19], [20], [21], [22], [23], [24], [25], [26], [27], [28], [29], [30], [31], [32],^48^,^49^]. As a result, this review investigated, assessed, and synthesised relevant research to determine the efficacy of reciprocal instruction and the characteristics that are linked to increased reading comprehension outcomes, academic achievement and learning outcomes [49]. For establishing and enhancing students' reciprocal teaching skills, reciprocal teaching has proven to be a highly effective learning technique. Throughout the reciprocal teaching technique, students' use of the four strategies: predicting, questioning, clarifying, and summarising enhanced their understanding and allowed them to grasp the most information from the given text. The reciprocal teaching technique, according to the results analysis, assisted poor readers in developing their reading skills by using predicting, clarifying, questioning, and summarising, which enabled them to interact with the text to construct meaning [19,25,34]. As a result, if you teach in the primary grades, you should employ reciprocal teaching in your classroom. According to Gazula et al. [56] studied the benefits and challenges of Reciprocal Peer Tutoring in health professional education only but our research focused on using reciprocal teaching to improved academic achievement in most of fields such as (English language, Arabic language, mathematics and science subjects). For establishing and refining students' reciprocal teaching skills, the reciprocal teaching approach has proven to be a very effective learning tool. It has been observed that students' usage of the four strategies—predicting, questioning, clarifying, and summarising—during the reciprocal teaching method improved their comprehension and allowed them to fully assimilate the information from the given material. By using strategies like anticipating, clarifying, asking questions, and summarising, which allowed them to interact with the text to create meaning, the reciprocal teaching technique helped weak readers improve their reading ability as well as their academic achievement.

Academic achievement and in-class learning are inextricably linked. Understanding learning processes also improves academic performance. Teachers must plan learning activities for their classes and promote intellectual conversation among students as well as learning dispositions that foster a sense of community and responsibility for one another [57]. These exercises could involve groups of students working together to solve issues by relying on one another and summarising, clarifying explanations with questions, speculating on potential solutions, and providing alternative approaches. Since many low achievers still lack the experiences that would provide them the background information essential to acquire some new things, it is important for teachers to recognise this fact. Teachers must evaluate the needs of their pupils and implement learning methods that are in line with those demands. Students don't seem to learn very much by simply listening to the teacher lecture, memorising prepared activities, and presenting their answers; instead, they need to articulate what they are learning, write about it, relate it to personal experiences, and use it in their everyday lives.

## 8 Recommendations

The use of reciprocal teaching is recommended to lecturers in colleges of education for the teaching of most courses in order to improve achievement, lecturers should encourage pre-service teachers to become familiar with reciprocal teaching so that it will be convenient and easy to use when practising as teachers, lecturers and supervisors should encourage pre-service teachers to use reciprocal teaching strategies in order to improve their students' achievement, lecturers and supervisors should encourage pre-service teachers to use reciprocal teaching strategies in order to improve their students' achievement, lecturers, the government should organise a type of in-service and re-training programme for teachers to learn how to use reciprocal teaching effectively strategies, to update instructors' knowledge of reciprocal teaching and learning, curriculum designers should add some collaborative teaching approaches. Since this would enhance their academic achievement, students should always be permitted to participate freely and actively in class with their classmates and teachers. Finally, the researchers recommended to study reciprocal teaching with technology (Internet Reciprocal Teaching method) because most of the educational fields nowadays using virtual learning environments, also the researchers suggested to application reciprocal teaching with eight techniques, including predicting, clarifying, questioning, picturing, linking, calculating, summarising, and providing feedback, because its suitable with mathematics, Physics and chemistry subjects to use it with higher thinking skills and meta-cognition skills.

## Author contribution statement

All authors listed have significantly contributed to the development and the writing of this article.

## Data availability statement

Data will be made available on request.

## Additional information

No additional information is available for this paper.

## Declaration of competing interest

The authors declare that they have no known competing financial interests or personal relationships that could have appeared to influence the work reported in this paper
